# Role of *Toxoplasma* IgA as Part of a Reference Panel for the Diagnosis of Acute Toxoplasmosis during Pregnancy

**DOI:** 10.1128/JCM.01357-18

**Published:** 2019-01-30

**Authors:** Tudor Rares Olariu, Brian G. Blackburn, Cindy Press, Jeanne Talucod, Jack S. Remington, Jose G. Montoya

**Affiliations:** a Toxoplasma Serology Laboratory, Palo Alto Medical Foundation, Palo Alto, California, USA; b Department of Medicine, Stanford University School of Medicine, Stanford, California, USA; c Division of Infectious Diseases and Geographic Medicine, Stanford University School of Medicine, Stanford, California, USA; d Victor Babes University of Medicine and Pharmacy, Timisoara, Romania; Boston Children's Hospital

**Keywords:** *Toxoplasma gondii*, toxoplasmosis

## Abstract

This study evaluated the usefulness of adding the Toxoplasma gondii IgA antibody enzyme-linked immunosorbent assay (ELISA) to the serologic panel of tests done for the diagnosis of acute toxoplasmosis in pregnant women in a reference laboratory in the United States. We conducted a retrospective study of 690 consecutive pregnant women with positive T. gondii IgG antibody test results who also had T. gondii IgA and IgM antibody tests performed. Patients were defined as acutely or chronically infected with T. gondii based on a panel of serologic tests performed at the Palo Alto Medical Foundation Toxoplasma Serology Laboratory (PAMF-TSL). Among the 81 women who were positive by T. gondii IgA antibody ELISA testing, 61 (75.3%) were acutely infected with T. gondii, while of the 547 who were negative by IgA testing, only 24 (4.4%) were acutely infected (*P* < 0.001). Among the 71 women who were positive by both IgA and IgM antibody tests, 61 (85.9%) were acutely infected, whereas 24 (19.2%) of the 125 women who were positive by only the IgM ELISA were acutely infected (*P* < 0.001). These results demonstrate that pregnant women with T. gondii IgA antibodies are more likely than pregnant women without T. gondii IgA antibodies to have had a recent infection with T. gondii. *Toxoplasma* IgA antibody testing can therefore improve the accuracy of a serologic panel for the diagnosis of acute toxoplasmosis during pregnancy. Physicians who ordered testing only for T. gondii IgG and IgM should also request additional testing for IgA and IgG avidity, if both IgG and IgM are positive. This further testing should, ideally, be performed in a reference laboratory.

## Abstract (precis)

This study evaluated the usefulness of adding the Toxoplasma gondii IgA antibody enzyme-linked immunosorbent assay (ELISA) to the serologic panel of tests done for the diagnosis of acute toxoplasmosis in pregnant women in a reference laboratory in the United States. We conducted a retrospective study of 690 consecutive pregnant women with positive T. gondii IgG antibody test results who also had T. gondii IgA and IgM antibody tests performed.

## INTRODUCTION

Toxoplasma gondii is a ubiquitous parasite that has the capacity to cross the placenta and infect the fetus when pregnant women contract a primary infection during pregnancy (1). The timing of infection during gestation should be determined, since the risk of transmission and the severity of disease depend upon the gestational age at which the maternal infection was acquired; the transmission risk is nearly zero if the infection was acquired prior to pregnancy (2). The optimal approach to this end is to routinely test each pregnant woman for T. gondii IgG and IgM antibodies as soon as pregnancy is recognized in order to identify T. gondii-seronegative women at risk for seroconversion (3). Seronegative women can then be periodically tested during pregnancy in order to institute immediate anti-*Toxoplasma* treatment to those diagnosed with acute infection. This approach has been successfully implemented in several countries, including France, Austria, Italy, and Slovenia (3, 4).

Because routine prenatal screening is not currently done in the United States, laboratories are usually expected to determine the timing of T. gondii infections during gestation from a single serum sample. In addition, interpretation of the results of currently available serologic methods for the diagnosis of acute infections in pregnant women is complicated by (i) the prevalence of T. gondii IgG antibodies in chronically infected women, (ii) the fact that T. gondii IgM antibodies may persist for a year or more after infection, and (iii) the fact that false-positive T. gondii IgM antibody results are not uncommon outside of reference laboratories (5, 6). Although the IgG avidity test has proved to be useful for the exclusion of acute T. gondii infection during the first 12 to 16 weeks of gestation (1), a need still remains for serologic methods that will further ascertain the diagnosis of acute infection from a single serum sample in pregnant women. To address these challenges, the *Toxoplasma* reference laboratory in the United States (Palo Alto Medical Foundation Toxoplasma Serology Laboratory [PAMF-TSL]) has implemented a battery of serologic tests aimed at determining whether *Toxoplasma* infections are acute or chronic and estimating the timing of such infections (7, 8). These standardized tests have been approved for clinical use in the United States as laboratory-developed tests (LDTs) and include the Sabin-Feldman dye test (DT; IgG); double-sandwich enzyme-linked immunosorbent assay (ELISA) for detection of IgM, IgA, and IgE antibodies; the differential agglutination (agglutination of acetone [AC]-fixed tachyzoites versus that of formalin [HS]-fixed tachyzoites) test (AC/HS test); and the IgG avidity assay (8, 9).

Although the usefulness of detecting T. gondii IgA antibodies for the serodiagnosis of toxoplasmosis in pregnant women is controversial (10), a number of investigators have found testing for T. gondii IgA antibodies to be helpful for the serodiagnosis of recent infections in pregnant women (11–13). We have previously described our use of a T. gondii IgA antibody test for the diagnosis of acute toxoplasmosis (14).

Because many of the PAMF-TSL serologic tests, including the IgA ELISA, the AC/HS assay, and the IgG avidity assay, were implemented subsequent to our previous report, we undertook an evaluation of the usefulness of adding the T. gondii IgA ELISA to the serologic panel of tests done at our U.S. reference laboratory, with the aim of diagnosing acute toxoplasmosis in pregnant women, for most of whom only a single serum sample was available.

## MATERIALS AND METHODS

We retrospectively reviewed serologic test results from 690 consecutive pregnant women who were positive for T. gondii IgG antibodies and who also had T. gondii IgA and IgM antibody tests performed; for a given patient, only the first serum sample submitted was used. Samples were obtained from the Toxoplasmosis Biobank, which is available at the Palo Alto Medical Foundation Toxoplasma Serology Laboratory (PAMF-TSL; Palo Alto, CA; available at www.pamf.org/serology/ [toxolab@pamf.org]). PAMF-TSL is a not-for-profit reference laboratory dedicated solely to the diagnosis of toxoplasmosis; the laboratory complies with all diagnostic laboratory regulations of CLIA and California, New York, and other state regulatory agencies.

### Serologic tests.

The serologic tests performed included the Sabin-Feldman dye test (DT) for T. gondii IgG antibodies (15), the T. gondii IgM ELISA (16), the T. gondii IgA ELISA (14), the differential agglutination (AC/HS) test (17, 18), and the Vidas IgG avidity test (bioMérieux, Marcy l'Etoile, France) (19). The DT, T. gondii IgM and IgA ELISAs, and the AC/HS test are LDTs performed with antigens and conjugates produced in-house using established procedures by qualified personnel. All reagents are validated for appropriate reactivity before use in diagnostic testing. Standards to generate a standard curve and three levels of controls are included on every microplate for the ELISAs. Multiple levels of controls are also included with each test run for the non-ELISAs.

The IgG avidity test is a commercial, FDA-approved test kit (bioMérieux Inc, Marcy l'Etoile, France). This test was validated on-site before initial use. The manufacturer’s instructions regarding calibration and the running of controls were followed. Each new lot and/or shipment of kits was also validated using well-characterized sera before use.

An in-house internal proficiency program is used at least annually to verify assay accuracy. In addition, an exchange program, using samples tested in a blind manner, actively takes place with other European laboratories. The test performance and quality assurance/quality control of the Toxoplasma Serology Laboratory have been reviewed and approved annually by CLIA, California, and New York inspectors during multiple site visits for more than 20 years, as have personnel competency monitoring policies and other components of the quality management system, which is based on CLSI recommendations.

### Interpretation of tests results.

The DT was considered positive at a titer of ≥1:16, the T. gondii IgM ELISA was considered positive at a titer of ≥2.0, and the T. gondii IgA ELISA was considered positive at a titer of ≥2.1. For the purposes of this study, high titers for T. gondii IgA and IgM antibodies were arbitrarily defined as a titer of ≥5 in both tests, and equivocal ELISA results were considered negative.

For the purposes of this study, AC/HS test interpretations included acute (may reflect a recent infection, although the infection can persist for months following the initial infection) and equivocal or nonacute (infection was acquired at least many months prior to the date of serum sampling) (17, 18). The Vidas IgG avidity test was interpreted (according to the manufacturer’s instructions) as follows: a low (<20%) or equivocal (between 20 to 29.9%) result indicates that primary infection within the past 4 months is possible, and a high (>30%) result excludes the possibility of a primary infection within the previous 4 months (19).

The selection of serologic tests performed on individual patients at PAMF-TSL is initially based upon the requesting physician’s order. Often, this consists of the complete panel of tests described above. However, in some patients, only testing for T. gondii IgG or IgM antibodies is initially ordered. After review of the initial results, further testing is sometimes performed at the recommendation of consulting physicians at PAMF-TSL. For example, if a patient’s AC/HS test result indicates a nonacute infection, the avidity test is not recommended by PAMF-TSL consultants unless other serologic test results suggest a recently acquired infection. If, however, the patient’s physician requests performance of the entire battery of serologic tests, the avidity and other tests are always performed.

To determine the frequency with which the T. gondii IgA ELISA antibody test was performed as part of preordered panels versus as a subsequent PAMF-TSL physician recommendation, we reviewed a 10% sample of T. gondii IgA antibody-negative patients (*n* = 50) and a 15% sample of T. gondii IgA antibody-positive patients (*n* = 14) to determine the indication for T. gondii IgA antibody testing.

### Definition of acute and chronic infection.

Because clinical information was not systematically available (and given that acute T. gondii infection is asymptomatic in most patients) (20), we used a serologic definition for acute infection. For the purposes of the study, acute T. gondii infection was defined in those pregnant women with a positive IgM ELISA result (≥2.0 units) and a low IgG avidity test result (<20.0%) and an acute pattern in the AC/HS test (17–19). For analyses that compared T. gondii IgA to T. gondii IgM antibodies for determination of infection timing, the T. gondii IgM antibody result was removed from the case definition of recent infection; for example, when analyzing the predictive value that T. gondii IgA antibody testing added to T. gondii IgM antibody testing, the definition for an acute infection with T. gondii included only an avidity below 20 plus an acute pattern in the AC/HS test; T. gondii IgM antibody test results were not part of the case definition. T. gondii infection was considered chronic in pregnant women who had either a negative T. gondii IgM antibody test result (<1.9 units) or a nonacute pattern in the AC/HS test or an IgG avidity of >30.0% (17–19). Women who did not fit the serologic definition for either acute or chronic infection were considered equivocal in terms of infection timing and were excluded from the analysis. Denominators for acute versus chronic infection thus differed according to the number of women who fit the classification for acute or chronic infection in various analyses. The PAMF Institutional Review Board approved our study of stored samples and associated laboratory information.

### Statistical analysis.

Data were compiled in a Microsoft Excel database, version 2011 (Microsoft Corp., Redmond, WA, USA), and univariate analyses were performed with the Epi Info statistical package, version 3.3.2 (Centers for Disease Control and Prevention, Atlanta, GA). The Mantel-Haenszel chi-square test and Fisher’s 2-tailed exact test were used to compare proportions between groups.

## RESULTS

### Testing performed.

The 690 pregnant women who were positive for T. gondii IgG antibodies and who also had T. gondii IgA and IgM antibody tests performed ranged in age from 14 to 55 years (mean = 32.2 years). The differential agglutination (AC/HS) test was performed for 685 (99.3%) of the 690 women, and the IgG avidity test was performed for 292 (42.3%). In 628 (91%) of the 690 women, it was possible to classify infection as acute or chronic according to our definitions. In cases in which the IgG avidity test was not performed, the diagnosis of an acute or chronic infection was based largely on the AC/HS test results.

### T. gondii IgA and IgM antibody positivity rates.

Of the 690 women, 90 (13.0%) had IgA antibodies and 258 (37.3%) had IgM antibodies. Eighty-nine percent of those with IgA antibodies also had IgM antibodies, whereas only 31.0% of women with IgM antibodies also had IgA antibodies. Women with IgA antibodies were more likely to have an acute AC/HS pattern (75.3% versus 39.6%; *P* < 0.001) and low avidity results (83.1% versus 63.9%; *P* = 0.001) than those with IgM antibodies alone (Table 1).

**TABLE 1:** Toxoplasma gondii IgA and IgM antibody test results compared to AC/HS and IgG avidity results in pregnant women^*a*^

Result(s) for individual serum samples (no. of samples)	No. (%) of women					
	AC/HS assay			Avidity assay		
	Total	A (%)	NA (%)	Total	L (%)	H (%)
IgA (+) (90)	89	67 (75.3)	22 (24.7)	89	74 (83.1)	15 (16.8)
IgA (−) (600)	502	44 (8.7)	458 (91.2)	203	74 (36.5)	129 (63.5)
IgM (+) (258)	237	94 (39.6)	143 (60.3)	194	124 (63.9)	70 (36.1)
IgM (−) (432)	354	17 (4.8)	337 (95.2)	98	24 (24.5)	74 (75.5)
IgM (+), IgA (+) (80)	79	62 (78.5)	17 (21.5)	79	69 (87.3)	10 (12.6)
IgM (+), IgA (−) (178)	158	32 (20.2)	126 (79.7)	115	55 (47.8)	60 (52.2)
IgM (−), IgA (+) (10)	10	5 (50)	5 (50)	10	5 (50)	5 (50)
IgM (−), IgA (−) (422)	344	12 (3.5)	332 (96.5)	88	19 (21.6)	69 (78.4)

a (+), positive; (−), negative; A, acute; NA, nonacute or equivocal pattern; L, low or equivocal; H, high.

Sera from women who were positive for both IgA and IgM antibodies more commonly had an acute AC/HS pattern (78.5%) than those who were negative for IgA antibodies but positive for IgM antibodies (20.2%) (*P* < 0.001). Sixty-nine (87.3%) of the 79 women who had both IgA and IgM antibodies had a low avidity test result, while 55 (47.8%) of the 115 who were negative for IgA antibodies but positive for IgM antibodies had a low avidity test result (*P* < 0.001) (Table 1).

The prevalence of IgA antibodies decreased with increasing avidity (Table 2). Among the 144 women with a high avidity test result, only 10.9% had IgA antibodies, whereas 48.6% had IgM antibodies (Table 2) (*P* < 0.001).

**TABLE 2:** Toxoplasma gondii IgA and IgM antibody test results stratified by IgG avidity results in 292 pregnant women^*a*^

Avidity	*n*	IgA		IgM	
		P (%)	OR^*b*^	P (%)	OR^*c*^
<5	23	18 (78.2)	1 (ref)	22 (95.6)	1 (ref)
5–9.9	46	30 (65.2)	0.521	41 (89.1)	0.373
10–14.9	28	12 (42.8)	0.208	18 (64.3)	0.082
15–19.9	20	9 (45)	0.227	19 (95)	0.864
20–29.9	31	5 (16.1)	0.053	24 (77.4)	0.156
>30	144	15 (10.9)	0.032	70 (48.6)	0.043

a *n*, number of women tested; P (%), number (percentage) of women positive; ref, reference.

b OR, odds ratio for IgA positivity compared to women with an IgG avidity test result of <5.

c OR, odds ratio for IgM positivity compared to women with an IgG avidity test result of <5.

### Acute and chronic infection.

Of the 628 women in whom it was possible to classify infection as acute or chronic, 85 (13.5%) were acutely infected and 543 (86.5%) were chronically infected. IgA antibodies were present in 61 of the 85 (71.7%) women who were acutely infected compared to 20 (3.7%) of the 543 with chronic infection (*P* < 0.001; odds ratio = 66.46; 95% confidence interval, 34.69 to 127.31). Among the women with IgA antibodies, 75.3% (61 of 81) were acutely infected, whereas only 4.4% (24 of 547) of those without IgA antibodies were acutely infected (*P* < 0.001) (Table 3). High T. gondii IgA antibody titers (≥5.0) were even more strongly predictive of recent infection: 31 (91.2%) of the 34 women with an IgA antibody titer of ≥5.0 had been acutely infected, whereas only 54 (9.1%) of 594 women with an IgA antibody titer of 4.9 or lower had been acutely infected (*P* < 0.001). The T. gondii IgA ELISA had a sensitivity of 71.8%, a specificity of 96.3%, a positive predictive value of 75.3%, and a negative predictive value of 95.6% for differentiating pregnant women who had acute T. gondii infection from those who had chronic infection. Among women with both IgA and IgM antibodies, 61 (85.9%) of 71 were acutely infected, whereas only 19.2% (24 of 125) of women who were IgA negative but IgM positive were acutely infected (*P* < 0.001).

**TABLE 3:** Toxoplasma gondii IgA antibody test results in pregnant women with acute and chronic infection^*a*^

IgA ELISA test results	No. (%) of women with:		
	Total	Acute infection	Chronic infection
Positive	81	61 (75.3)	20 (24.7)
Negative	547	24 (4.4)	523 (95.6)
Total	628	85	543

a Odds ratio = 66.46 (95% confidence interval, 34.69 to 127.31).

Ten women were IgA antibody positive but IgM antibody negative. There was a trend toward these women having been recently infected (40.0%, 4 of 10) more often than IgA antibody-negative and IgM antibody-positive women (19.2%, 24 of 125; *P* = 0.11).

### Indication for T. gondii IgA antibody testing.

In the 10% sample of T. gondii IgA antibody-negative women reviewed for testing indication, the T. gondii IgA antibody test was performed as part of the serologic panel (at the ordering physician’s request) for 44 (88%) of 50 women and at the request of PAMF-TSL for the remaining 12%. In the 15% sample of T. gondii IgA antibody-positive women reviewed in this manner, the T. gondii IgA antibody test was performed as part of the panel (at the ordering physician’s request) for 4 (29%) of 14 women and at the request of PAMF-TSL for the remaining 71%.

## DISCUSSION

Our study demonstrates that the presence of T. gondii IgA antibodies in pregnant women, in the context of a serologic panel available at PAMF-TSL, significantly increases the chances that T. gondii infection was acute compared to patients without T. gondii IgA antibodies. Several investigators who monitored patients after acute infection with T. gondii have reported a high prevalence of T. gondii IgA antibodies in these populations (7, 21–24). Some authors have found that T. gondii IgA testing is controversial and is either too insensitive or reactive for too long, suggesting that IgA is not a dependable marker for a recently acquired *Toxoplasma* infection (10, 25). However, similar to the findings reported here, a number of investigators have found that T. gondii IgA is helpful for the diagnosis of recent infection during pregnancy (11–13). These authors detected IgA antibodies directed against P30 in the sera of all patients during the acute phase of toxoplasmosis (11, 12). In the majority of their cases, when the level of IgG continued to rise and IgM antibodies persisted, IgA antibodies disappeared earlier and were not detected in the chronic phase of toxoplasmosis (11, 12). In addition, parallel testing for T. gondii IgA and IgM antibodies has been considered valuable by other investigators for diagnosing a recently acquired T. gondii infection, especially during pregnancy (11, 12, 26).

Importantly, pregnant women who were positive for T. gondii IgA antibodies in our study were 17 times more likely to have been recently infected with T. gondii than those who had negative T. gondii IgA antibody results. The higher rates of positivity for T. gondii IgM than IgA in chronically infected pregnant women suggest that T. gondii IgA antibodies may not persist as long as T. gondii IgM antibodies in these patients. Because T. gondii IgM antibodies can persist for prolonged periods after acute infection (27), their presence is not necessarily indicative of a recent infection (28). Our data demonstrate that women with both T. gondii IgA and IgM antibodies were four times as likely to have had a recent infection as those in whom only IgM antibodies were present.

Given these data, adding T. gondii IgA antibody testing to the existing serologic panel available at PAMF-TSL improved detection of recent *Toxoplasma* infections. It is important to note that T. gondii IgA antibodies will not always be present early in acute infection, as seen in the 4.4% of the women in our series who were T. gondii IgA negative, despite having acute infections. In addition, the frequency of acutely infected individuals with negative T. gondii IgA antibody test results is likely to be greater than 4.4% since serum samples sent to PAMF-TSL are likely to have a bias favoring patients with a higher probability of an acute infection. As a consequence, while a negative T. gondii IgA ELISA result makes recent T. gondii infection less likely, it does not exclude a recent infection (20).

An inverse correlation also existed between the value of the IgG avidity test results and the presence of T. gondii IgA antibodies, suggesting that the more recently the infection occurred, the more likely it is that the IgA test result will be positive. In contrast, women who had a high avidity result (indicating that the infection occurred at least 4 months earlier) were significantly less likely to have a positive T. gondii IgA antibody test result. As previously reported by Roberts et al. (29), this suggests that in the population of women with a chronic infection that we studied, T. gondii IgA antibodies persisted only rarely (whereas T. gondii IgM antibodies persisted nearly half the time). In addition, the magnitude of the T. gondii IgA antibody response seems to be an important predictor, given that patients with high T. gondii IgA antibody titers were even more likely to have been recently infected.

We were surprised to find that 10 women who were T. gondii IgA ELISA antibody positive were T. gondii IgM antibody negative. In six of them, IgG avidity or AC/HS test results excluded acute infection, suggesting that in some cases, the T. gondii IgA antibody response may persist longer than the T. gondii IgM antibody response. When it occurs, the persistence of T. gondii IgA (or IgM) antibodies in chronic infections may be explained by factors such as patient heterogeneity, infecting parasite stage (oocyst versus cyst), immune status at the time of blood collection, time of blood sampling relative to the time of onset of infection, assay technique (25), or parasitemia during cases of serologic reactivation (22). The other four patients had avidity and AC/HS test results which suggested that a recent infection may have been present; these negative T. gondii IgM antibody test results may have occurred because testing was performed shortly after T. gondii IgM antibody titers decreased or these patients may be similar to those reported recently by European investigators, namely, acutely infected patients negative for T. gondii IgM antibodies (30). Because only a single serum sample is usually submitted for testing in the United States, the possibility of recent infection in women with positive T. gondii IgA antibody and negative T. gondii IgM antibody test results cannot be excluded.

**FIG 1:**
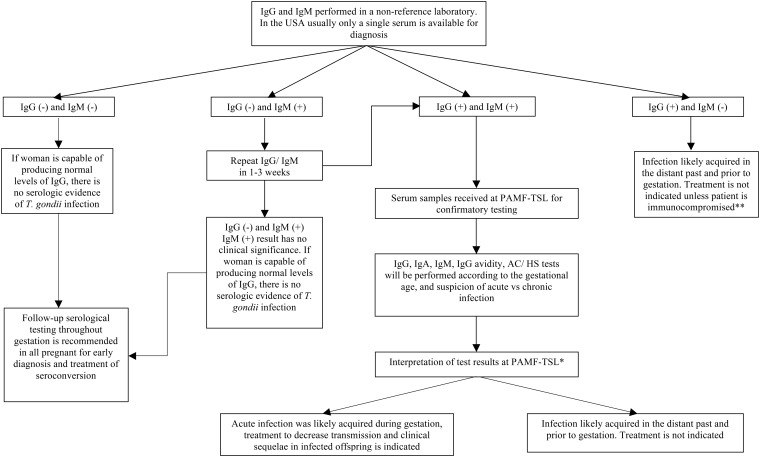
Testing algorithm for the serologic diagnosis of toxoplasmosis in pregnant women in the United States. *, a final interpretation of the serologic panel is made after evaluation of each test result, including titer level and a combinatorial approach where all test results are taken into account. Subsequently, gestational age, immune status, and the woman’s other medical history are factored into providing recommendations regarding treatment. **, serum samples from immunocompromised patients suspected of having chronic infection should be sent to PAMF-TSL for further confirmatory testing and interpretation.

In clinical practice, physicians who ordered testing only for T. gondii IgG and IgM should also request additional testing for T. gondii IgA and IgG avidity only if both IgG and IgM are positive. This additional testing with IgA and IgG avidity should, ideally, be performed in a reference laboratory, and the results should be interpreted by an experienced specialist who is familiar with this reference serologic panel (Fig. 1).

Our study has limitations, including the retrospective study design, the lack of clinical information, and the fact that serum samples were obtained in the United States, where there is a lack of systematic testing for toxoplasmosis (including T. gondii IgA antibodies), which may have resulted in ascertainment bias. It is possible that because of this, women who had T. gondii IgA antibody testing done were more likely *a priori* to be T. gondii IgA antibody positive (and to have been recently infected), given that most of the T. gondii IgA antibody-positive patients in our cohort had T. gondii IgA antibody testing performed at the request of PAMF-TSL consultants after an initial review of other elements of a patient’s *Toxoplasma* serologic profile. Most such patients therefore had other serologic results, including detectable T. gondii IgM antibodies, that suggested acute infection, and this might have resulted in an overestimation of the prevalence of acute infection in women with T. gondii-specific IgA antibody compared to that in an unbiased population.

Our results indicate that in pregnant women, positive T. gondii IgA antibody results make a recent infection more likely. T. gondii IgA antibody testing might therefore represent a valuable adjunct to the PAMF-TSL serologic panel (dye test IgG, IgM, IgA, IgE, AC/HS, IgG avidity) used for determining the timing of the infection in pregnant women.
